# Unrevealing the Interaction Between Electrode Degradation and Bubble Behaviors in an Anion Exchange Membrane Water Electrolyzer

**DOI:** 10.1002/advs.202412962

**Published:** 2025-02-07

**Authors:** Lizhen Wu, Qing Wang, Shu Yuan, Xiaohan Mei, Qian Wang, Xiaohong Zou, Kouer Zhang, Xiaoyu Huo, Xingyi Shi, Zhefei Pan, Xiaohui Yan, Liang An

**Affiliations:** ^1^ Department of Mechanical Engineering The Hong Kong Polytechnic University Hung Hom Kowloon Hong Kong SAR 26680 China; ^2^ Institute of Fuel Cells School of Mechanical Engineering Shanghai Jiao Tong University 800 Dongchuan Road Shanghai 200240 China; ^3^ Institute of Engineering Thermophysic School of Mechanical Engineering Shanghai Jiao Tong University 800 Dongchuan Road Shanghai 200240 P. R. China; ^4^ Institute of Engineering Thermophysics School of Energy and Power Engineering Chongqing University Chongqing 400044 P. R. China; ^5^ Research Institute for Advanced Manufacturing The Hong Kong Polytechnic University Hung Hom Kowloon Hong Kong SAR 26680 China

**Keywords:** AEMWE, bubble behaviors, durability, multi‐scale visualization, stainless steel felt

## Abstract

Stainless steel felt has been employed in AEMWE as a combination of oxygen evolution reaction (OER) electrocatalysts and porous transport layers, which are not only easy to prepare but also have excellent OER activity under alkaline conditions. However, by realizing detailed electrochemical analysis and multi‐scale visualization of the bubble behaviors, it is found that the combined effect of chemical and electrochemical corrosion led to the constant accumulation of metal oxides on the stainless steel fiber surface post‐durability compared to the slow‐growing hydroxides after initial activation. Moreover, the rougher fiber surface morphology and weaken hydrophilicity cause the adjacent bubbles are slower to detach from the electrode and are more likely to fusion. The measured diameter of bubbles leaving the electrode almost doubles, while the total number of bubbles decreases by about two‐thirds, causing the increase of plug flow in the flow field and deteriorating the performance and long‐term stability of AEMWE.

## Graphical abstract

Stainless steel felt in AEMWE exhibits excellent OER activity, but its durability is compromised by chemical and electrochemical corrosion, leading to metal oxide accumulation that alters fiber morphology and hydrophilicity. Consequently, bubble detachment slows, resulting in larger bubbles and decreased quantity, which increases plug flow in the flow field and diminishes performance and stability.

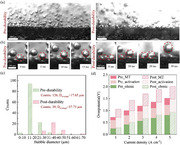

## 1 Introduction

Water electrolysis is recognized as a promising strategy to achieve net‐zero CO_2_ emissions in the future, primarily because of integrating with solar and wind systems and utilizing renewable electricity.^[^ ^1^, ^2^, ^3^ ^]^ Additionally, it relies on an abundant reactant (water) and produces pure and green hydrogen.^[^ ^4^, ^5^, ^6^ ^]^ Among the various water electrolysis technologies, the anion exchange membrane water electrolyzer (AEMWE) stands out for its ability to combine the advantages of both alkaline water electrolyzer (AWE) and proton exchange membrane water electrolyzer (PEMWE). AEMWE can operate at high efficiency with lower costs, representing a promising direction for sustainable and green hydrogen production.^[^ ^7^, ^8^, ^9^ ^]^ In this work, a stainless steel felt is adopted as the anode electrode for AEMWE, which has been shown to not only eliminate the need to prepare a conventional catalyst layer made of ionomer and nanoparticle catalysts but also to have a superior oxygen evolution reaction (OER) activity under alkaline conditions.^[^ ^10^, ^11^, ^12^ ^]^

As a combined OER electrocatalyst and porous transport layer,^[^ ^13^, ^14^ ^]^ Chen et al.^[^ ^14^ ^]^ found that stainless steel felts exhibited better performance and stability in zero‐gap AEMWE compared to conventional electrodes consisting of iridium (Ir) and ionomer. Tricker et al.^[^ ^15^ ^]^ also demonstrated that a mixture of iron and nickel hydroxyl oxides was formed on the surface of the electrode compared to untreated stainless steel electrodes. The mixture not only enhanced the OER activity but also surface hydrophilicity which is beneficial for the bubbles transport.^[^ ^16^, ^17^ ^]^ Notably, bubble dynamics including bubble evolution and bubble transport occur in this only key component. Hence, bubbles can be dramatically affected by changes in the surface characteristics of the combined electrode.^[^ ^18^ ^]^ If serious bubble clogging appears, they will increase the activation and mass transport overpotentials and make the electrode operate at a higher potential, accelerating the electrochemical corrosion and electrode degradation of AEMWE.^[^ ^15^, ^19^, ^20^ ^]^ It should be noted that the interaction between electrode degradation and bubble behaviors in water electrolyzers before and after long‐term operation has not been evaluated comprehensively.

To build the relationship between electrode degradation and bubble behaviors in AEMWE, we employed a combination of electrochemical techniques, characterization analysis, and multi‐scale bubble visualization. Our findings reveal that the surfaces of stainless steel fibers initially experience an increase in hydroxide formation during long‐term operation. Over time, a combination of chemical and electrochemical corrosion leads to the constant accumulation of metal oxides, resulting in a rougher surface morphology. Consequently, contact angle tests indicate a significant reduction in the hydrophilicity of the stainless steel felt. Furthermore, a high‐speed camera combined with a microscope has been utilized to observe in situ millimeter‐scale two‐phase flow in the flow field, as well as micrometer‐scale bubble behaviors at the electrode surfaces. The results indicate that an increase of plug flow in the flow field diminishes the effective water flow area directed toward the electrodes post‐durability. This phenomenon can be attributed to the slower detachment and easier merging of adjacent bubbles at the electrode surfaces, resulting from the roughened and weaken hydrophilic fiber surface. Notably, the measured diameter of bubbles detaching from the electrode post‐durability nearly doubled, while the total number of bubbles post‐durability decreased by approximately two‐thirds.

## 2 Results and Discussion

### 2.1 Electrochemical Analysis

**Figure 1:**
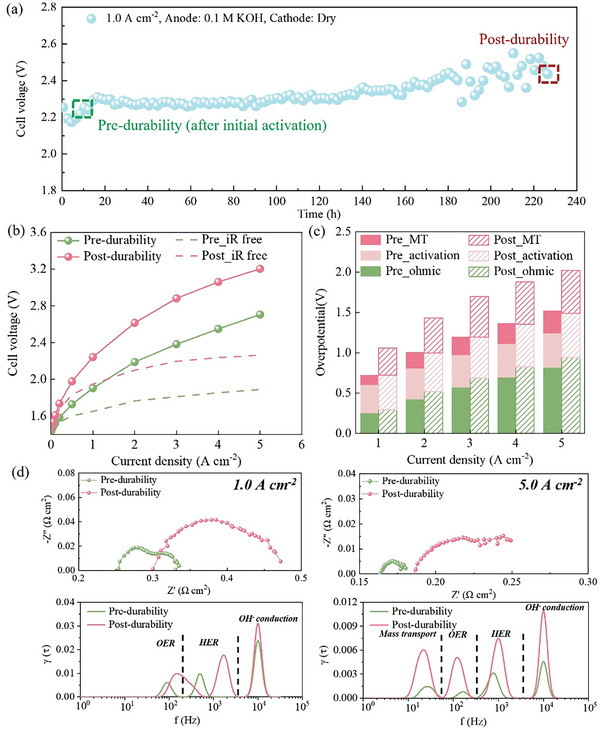
a) Durability of AEMWE using stainless steel electrode at 1.0 A cm^−2^. b) polarization and iR‐free curves for AEMWE pre‐ and post‐durability testing. c) Breakdown of each overpotential at different current densities. d) DRT analysis at 1.0 A and 5.0 A cm^−2^.

**Figure 2:**
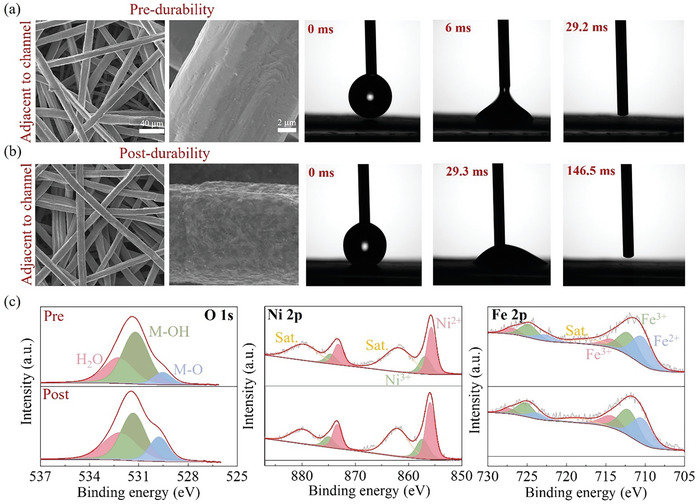
SEM image and contact angle testing: a) pre and b) post‐durability. c) XPS spectra of O 1s, Ni 2p, and Fe 2p.

**Figure** **1**a shows the 225‐h durability testing of AEMWE operating with a 0.1 m KOH solution feeding into the anode inlet at a rate of 5.0 mL min^−1^ at 80 °C. The setup for durability testing is shown in Figure S1 (Supporting Information). Figure 1b displays the polarization and iR‐free curves pre‐ and post‐durability of AEMWE operating with an only replaced 1.0 m KOH solution. It is worth noting that the pre‐durability stainless steel felt is not pristine but has been conditioned for ≈5–10 h. The results show that the electrochemical performance pre‐durability is significantly better than that under each current density. Figure 1c presents the breakdown of each overpotential at different current densities by using the Tafel‐approximation method.^[^ ^21^, ^22^ ^]^ It can be observed that the mass transport overpotential exhibits the largest increase (more than 100% at all current densities). And with the elevated current density, ohmic overpotential has the smallest increase (<15%). The ohmic overpotential is dependent of the internal resistance of AMEWE. Surface roughness increase of stainless steel fiber (Figure 2a,b) and membrane degradation (decreasing conductivity) as shown in Figure S2 (Supporting Information) increase the internal resistance, which can be proved by high‐frequency resistance (HFR) from EIS in Figure S3b (Supporting Information). The activation overpotential is a combination of hydrogen evolution reaction (HER) and OER, which is different with that in PEMWE (cathode side can be used as pseudo reference), so the activation overpotentials of HER and OER cannot be separated. Alternatively, distribution of relaxation times (DRT) analysis^[^ ^23^ ^]^ has been used in fuel cells successfully^[^ ^24^ ^]^ and is considered to be an effective tool to supplement a more detailed breakdown of all overpotentials in AMEWE, as shown in Figure 1d. The DRT analysis generally reveals four peaks, which can be sequenced from low to high frequency as follows: the mass transport process, the OER process, the HER process, and the OH^−^ conduction process, respectively. By increasing the current density from 1.0 to 5.0 A cm^−2^, not only the increase in activation impedance about OER is greater than that about HER, but also there is an additional peak regarding mass transport in the post‐durability case. A related three‐electrode test has been conducted to further demonstrate that the electrodes after durability presented worse performance, as shown in Figure S5 (Supporting Information). Since KOH solution only flows through the anode, it can be inferred that the change of mass transport overpotential is primarily influenced by the bubble clogging inside pre‐ and post‐durability electrodes. To further confirm our conclusion, we conducted an additional experiment to determine the extent of cathode electrode degradation. Change in Tafel slope can be used to show the change of activation overpotential at different current densities.^[^ ^25^ ^]^ By comparing the Tafel slope of pre‐ and post‐durability ((Figure S3 (Supporting Information): 46.5–72.93 mv dec^−1^, Δ1 = 26.43 mv dec^−1^), Case A (new anode // new membrane // post‐durability cathode) and case B (new anode // new membrane // new cathode) (Figure S4 (Supporting Information): 51.79–44.67 mv dec^−1^, Δ2 = 7.12 mv dec^−1^). The change in Tafel slope (Δ1 > Δ2) indicates that the increase in activation overpotential at the anode is 2.71 ((26.43–7.12)/ 7.12) times the increase in activation overpotential at the cathode. As for the mass transport overpotential, by comparing the increase in mass transport overpotential pre‐ and post‐durability (Figure 1c: 0.28–0.53 V at 5.0 A cm^−2^, Δ3 = 0.25 V), case A (new anode//new membrane//post‐endurance cathode), and case B (new anode//new membrane//new cathode) (Figure S4 (Supporting Information): 0.17–0.21 V at 5.0 A cm^−2^, Δ4 = 0.04 V). The increase in mass transport overpotential indicates that the increase in mass transport overpotential at the anode is ≈5 ((0.25–0.04)/ 0.04) times greater than the increase in mass transport overpotential at the cathode. Overall, there is a 2.71‐time increase in activation overpotential and a 5‐time increase in mass transport overpotential at the anode compared to the cathode, both of which indicate that the degradation of the anode and cathode are comparable, but anode stainless steel electrode degraded more severely.

### 2.2 Electrode Characterization

Scanning electron microscopy (SEM) and contact angle tests are necessary to directly reflect how the surface of stainless steel fiber affects its hydrophilicity. According to the SEM image and imbibition process of the water droplet comes into contact with the stainless steel felt in **Figure** **2**a,b, the surface of fiber post‐durability exhibits the rough surface of fibers compared with the pre‐durability electrode, which reduces its ability to absorb droplets by a factor of four (diminished surface hydrophilicity). It has been demonstrated that compared to the pristine stainless steel electrodes, the surface of stainless steel fibers post‐durability will have increased the mixture of iron and nickel hydroxyl oxides, contributing to the enhancement of OER activity and hydrophilicity,^15^ which can also be proved in the initial performance enhancement from Figure 1a. However, hydroxides are not always increased significantly after the initial conditioning. Over time, a combination of chemical and electrochemical corrosion leads to a continuous accumulation of metal oxides. Through the X‐ray photoelectron spectroscopy (XPS) in Figure 2c, it can be found that the XPS spectra of O 1s display peaks at 529.74,531.32, and 532.27 eV,^[^ ^26^, ^27^, ^28^ ^]^ corresponding to the oxides (M─O), hydroxides (M─OH) and H_2_O, respectively. Among them, the peak intensity of oxides (M─O) showed a significant increase. As for the XPS spectra of Ni pre‐ and post‐durability, it did not change significantly, while the XPS spectra of Fe pre‐ and post‐durability exhibited an increase in Fe^3+^. It can be inferred that the rough surface of fiber post‐durability mainly consists of metal oxides, and the obvious reddish brown color (Fe_2_O_3_) can also be seen in Figure S2 (Supporting Information).

### 2.3 In Situ Multi‐Scale Visualization

The percentage of various two‐phase flow regimes in the channel has been introduced to quantify the different kinds of bubble content. This index is defined as the average of the ratios of the area occupied by bubbles in each section of the channel to the total area of that section. It is mathematically represented by Equation (1):^[^ ^29^, ^30^ ^]^

(1) $$\mathrm{Bubble}\;\mathrm{Content}\;\mathrm{Index}=\Sigma \;\frac{\mathrm{volmue}\;\mathrm{of}\;\mathrm{each}\;\mathrm{channel}\;\mathrm{that}\;\mathrm{is}\;\mathrm{occupied}\;\mathrm{by}\;\mathrm{different}\;\mathrm{kinds}\;\mathrm{of}\;\mathrm{bubble}\;\mathrm{content}}{\mathrm{total}\;\mathrm{volume}\;\mathrm{of}\;\mathrm{each}\;\mathrm{channel}}\;\times \;100\;\%$$

**Figure 3:**
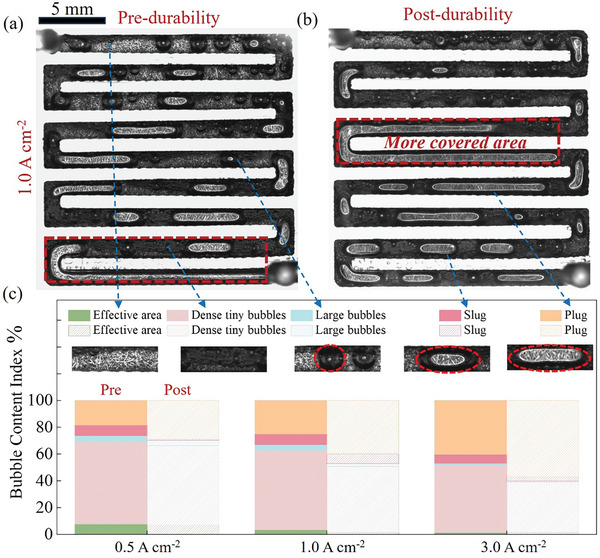
Millimeter‐scale visualization of two‐phase flow in flow field at 1.0 A cm^−2^: a) pre‐durability, b) post‐durability. c) Distribution of different flow regimes in the flow field.

The two‐phase flow regimes are generally categorized into effective zones, large bubbles, and slug and plug flow according to the severity of the bubble‐clogging phenomenon.^[^ ^31^ ^]^ In addition to these, dense tiny bubbles regime is an occurrence observed in KOH‐supplied AEMWE, which differentiates it from the pure water‐supplied PEMWE and is therefore additionally defined. For example, **Figure** **3**a,b illustrate the two‐phase flow regimes distribution in the flow field pre‐durability and post‐durability at 1.0 A cm^−2^. The two‐phase flow regimes at other current densities are presented in Figure S6 (also see videos S1 and S2, Supporting Information). Bubbles clogging in the flow field is clearly visible and worse after durability. We employ a quantitative analysis method to elucidate the changes in the two‐phase flow regimes. As shown in Figure 3a,b, the plug flow post‐durability covers more area that for the water supply to the electrode than that in pre‐durability. In detail, from Figure 3c, it is evident that there is minimal difference in the proportions of effective area and large bubbles flow when comparing pre‐and post‐durability across all current densities. However, there is a remarkable increase in the proportion of slug and plug flow post‐durability compared to pre‐durability, particularly noticeable at higher current densities (for two‐electrode cells) such as 3.0 cm^−2^.^[^ ^8^ ^]^ In addition, a significant decrease in the proportion of dense tiny bubbles flow can be seen post‐durability. Therefore, it can be hypothesized that dense tiny bubbles flow turns into slug and plug flow as the current density increases. Moreover, bubbles can cover the electrodes and affect the mass transport of water, as evidenced by the increase in cell voltage in Figure 1a. In addition, large bubbles in the post‐durability electrode cause uneven distribution of bubbles within it and uneven water transport to the electrode, which may be the main reason for the fluctuation of the cell voltage after 180 h in Figure 1a.

**Figure 4:**
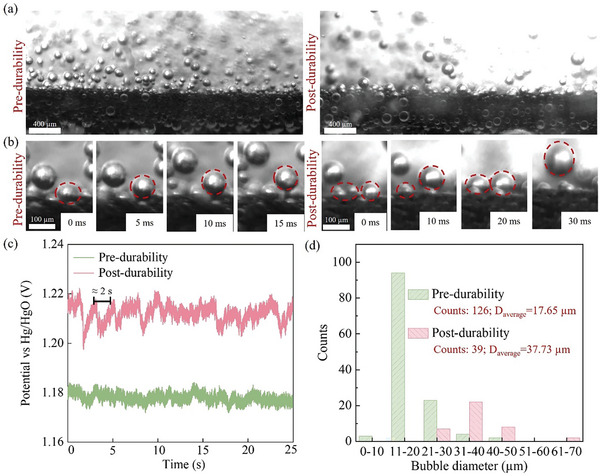
Micrometer‐scale visualization of bubble behaviors in pre‐ and post‐ durability electrode: a) 400 µm scale, b) 100 µm scale. c) Time transition of potential vs Hg/HgO at 0.5 A cm^−2^. d) The distribution of bubble sizes counted in high‐speed camera images in pre‐ and post‐ durability electrodes.

**Figure 5:**
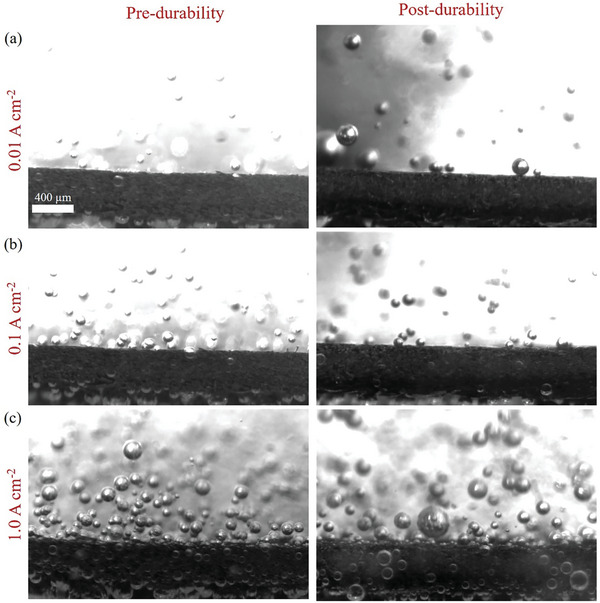
Micrometer‐scale bubble behaviors on the electrode surface pre‐ and post‐durability at current densities of 0.01, 0.1 and 1.0 A cm^−2^.

It is necessary to explore how the stainless steel electrode degradation affects the bubble clogging in the flow field. A high‐speed camera combined with a microscope has been utilized to observe in situ micrometer‐scale bubble behaviors at the electrode surfaces in a three‐electrode system, as shown in Figure S7 (Supporting Information). **Figure** **4**a,b display the bubble behaviors at the electrode pre‐ and post‐durability at various scales (also see videos S3, S4, Supporting Information). We mentioned earlier that metal oxides on the surface of stainless steel fibers present a rough morphology as well as weaken hydrophilicity, which can lead to adjacent bubbles not detaching from the electrodes in time and fusing easily. Moreover, the bubble tracking tool (Figure S8, Supporting Information) was used to quantify the diameter and number of detached bubbles. Notably, the measured diameter of detached bubbles almost doubled, while the total number of bubbles after durability decreased by about two‐thirds, as shown in Figure 4d. This phenomenon is not apparent at low current densities and is only visually observed with increasing current density, as shown in **Figure** **5**. At a current density of 0.5 A cm^−2^ (high current densities for three‐electrodes cell),^[^ ^32^ ^]^ the electrode potential fluctuation post‐durability was higher in amplitude but lower in frequency because of the undetached bubbles covering the active sites.

## 3 Conclusion

In this work, we used stainless steel felt as an anode electrode of AEMWE for durability tests. The results revealed that the stainless steel electrode post‐durability showed significant electrode degradation compared with that pre‐durability, mainly in the form of increased mass transport overpotential (more than 100%). The reason was attributed to the fact that hydroxides friendly to OER activity and hydrophilicity were not always increased, and the combined effect of chemical and electrochemical corrosion resulted in the continuous accumulation of metal oxides. As a result, the surface morphology of the stainless steel fiber becomes rougher and its hydrophilicity is significantly weakened (water droplets enter the electrode four times slower). In order to establish a direct relationship between electrode degradation and bubble behaviors in AEMWE, we also employed a combination of high‐speed camera and microscope to visualize millimeter‐scale two‐phase flow in the flow field as well as micrometer‐scale bubble behavior at the electrode surface. The results demonstrated that the increase of plug flow in the flow field reduces the effective water flow area toward the electrode post‐durability. This phenomenon can be attributed to the rough and less hydrophilic surface of the fibers, resulting in slower bubble detachment and easier fusion of adjacent bubbles. It is noteworthy that the measured diameter of bubbles detached from the electrode almost doubled, while the total number of bubbles post‐durability decreased by about two‐thirds. Potential fluctuations post‐durability showed higher amplitude but lower frequency. These findings highlight the need for an appropriate and efficient treatment of combined stainless steel electrodes to further improve the performance and long‐term stability of the AEMWE.

## Conflict of Interest

The authors declare no conflict of interest.

## Supporting information



Supporting Information

Supplemental Movie 1

Supplemental Movie 2

Supplemental Movie 3

Supplemental Movie 4

## Data Availability

The data that support the findings of this study are available from the corresponding author upon reasonable request.
