# Supplementary material for: Unrevealing the Interaction Between Electrode Degradation and Bubble Behaviors in an Anion Exchange Membrane Water Electrolyzer
Source: Adv Sci (Weinh). 2025 Feb 7;12(12):2412962. doi: 10.1002/advs.202412962 (PMC11948003; doi:10.1002/advs.202412962)
Supplement: Supplementary file 1 — Supporting Information [file ADVS-12-2412962-s003.docx]

**Unrevealing the Interaction between Electrode Degradation and Bubble Behaviors in an Anion Exchange Membrane Water Electrolyzer**

Lizhen Wu^a^, Qing Wang^a^, Shu Yuan^c^, Xiaohan Mei^e^, Qian Wang^e^, Xiaohong Zou^a^, Kouer Zhang^a^, Xiaoyu Huo^a^, Xingyi Shi^a^, Zhefei Pan^d,*^, Xiaohui Yan^c,*^, Liang An^a,b,*^

^a^ Department of Mechanical Engineering, The Hong Kong Polytechnic University, Hung Hom, Kowloon, Hong Kong SAR, China

^b^ Research Institute for Advanced Manufacturing, The Hong Kong Polytechnic University, Hung Hom, Kowloon, Hong Kong SAR, China

^c^ Institute of Fuel Cells, School of Mechanical Engineering, Shanghai Jiao Tong University, 800 Dongchuan Road, Shanghai 200240, China

^d^ Institute of Engineering Thermophysics, School of Energy and Power Engineering, Chongqing University, Chongqing 400044, China

^e^ Institute of Engineering Thermophysic, School of Mechanical Engineering, Shanghai Jiao Tong University, 800 Dongchuan Road, Shanghai 200240, PR China

*Corresponding authors.

E-mail: zhefei.pan@cqu.edu.cn (Zhefei Pan), [yanxiaohui@sjtu.edu.cn](mailto:yanxiaohui@sjtu.edu.cn) (Xiaohui Yan), [liang.an@polyu.edu.hk](mailto:liang.an@polyu.edu.hk) (Liang An)

**Experimental section**

***Materials***

The experimental materials include 316L stainless steel felt from Fuershun Electronic Materials and carbon paper from Hohsen Co. (Japan); Pt/C catalyst from Johnson Matthey (UK); PiperION A membrane (20 µm) and Nafion ionomer (D520, 5 wt %) from Fuel Cell Store (USA).

***Preparation of anode stainless steel electrode and cathode Pt/C electrode***

Stainless steel felts were cut into 2.0 cm × 2.0 cm and were cleaned in an ultrasonic bath with ethanol and DI water for 15 minutes, respectively. The cathode catalyst loading of anode is 0.5 mg cm^−2^ and the catalyst ink was prepared by mixing 60 wt % Pt/C with 5 wt % Nafion D520 at a mass ratio of 8:2, and then ethanol was added into the solution as a solvent.^[1]^ Subsequently, the ink was subjected to dispersion within an ultrasonic ice bath for 30 min.^[2]^ Thereafter, the ink was sprayed onto the carbon-based substrates and dried repeatedly, until the catalyst loading reached the desired value.^[3]^

***Characterization of anode stainless steel electrodes pre- and post-durability***

The morphology of anode stainless steel fibers pre- and post-durability were characterized using scanning electron microscopy (SEM) (Tescan VEGA3, Czech). X-ray photoelectron spectroscopy (XPS) analysis was performed using an X-ray photoelectron spectrometer (Thermo Scientific Nexsa, USA) with aluminum Kα radiation as the exciting source.^[4,5]^ The liquid water contact angle test is used to characterize how quickly liquid water enters the stainless steel felt pre- and post-durability.

***Preparation of membrane electrode assemblies***

A membrane electrode assembly (MEA) was created by combining a pair of anode stainless steel felt and cathode Pt-based carbon paper electrodes with a pre-treated PiperION A membrane (20 µm). The area of the electrode was then cut to 2.0 cm × 2.0 cm. For the membrane, the square PiperION A (20 µm) were cut into 2.5 cm × 2.5 cm small pieces and were immersed in 1.0 M KOH solution at ambient temperature for 24 h before being incorporated into the MEA. ^[6]^

***AEMWE fabrication and assembly***

The AEMWE was constructed using a home-made fixture with serpentine flow fields. They are made of nickel (anode) and titanium (cathode), with a depth of 1.0 mm each.^[7]^ In order to prevent electrolyte leakage, silicone gaskets with a thickness of 0.3 mm were placed between the two half fixtures. The cell was clamped by anode and cathode stainless steel end plates with four equally distributed bolts (3.0 N·m torque in each bolt).

***Electrochemical measurements***

During the measurement, the AEMWE was heated and maintained at 80 °C. A peristaltic pump (BT100-2J, LongerPump) was used to continuously supply 1M KOH solution into the anode inlet of the AEMWE at a mass flow rate of 5 ml min^-1^.^[8]^ Before testing, the cathode and anode electrodes are soaked in fresh 1M KOH for 1 hours.^[6]^ The cell was subjected to a conditioning process to ensure the performance is stable and reliable.^[9]^ For electrochemical measurements, an Autolab PGSTAT302N electrochemical workstation with an add-on booster was used to characterize the performance of the AEMWE. Firstly, we measured polarization curves over a current density range of 0 A cm^-2^ to 5.0 A cm^-2^. The PEMWE operated for 100 seconds at each current density to reach steady state and the cell voltage was determined by averaging the value of voltage for the last 10 seconds at each operating set point. Then, we performed the electrochemical impedance spectroscopy (EIS) measurements from 40 kHz - 10 Hz. The HFR obtained from the high frequency intercept of the Nyquist plot with the real axis was used to determine the ohmic resistance $R_{\text{ohmic}}$ of AEMWE pre- and post-durability. For durability testing, 0.1 M KOH solution was used instead of 1 M KOH solution to feed the anode. The electrolyte was replenished with an amount of water every 24 hours to maintain a constant KOH concentration during the experiment. After the durability experiment, the cell was directly conducted the electrochemical measurements mentioned above. Current was supplied using an KUAIQU DC power supply and the cell voltage was recorded every 2 hours using a paperless recorder.

Through obtaining the known data as described above, we can calculate the other overpotentials using the Tafel approximation,^[10–12]^ which is a very effective method that has been used to determine the mass transport overpotential of AEMWE. The relative equations are as follows:

$E_{\text{cell}}=E_{\text{rev}}+\eta_{\text{activation}}+\eta_{\text{ohmic}}+\eta_{\text{mass transport}}$ (1)

where $E_{\text{cell}}$(V) is the measured cell voltage, $E_{\text{rev}}$(V) is the reversible voltage, $\eta_{\text{activation}}$(V) is the activation overpotential, $\eta_{\text{ohmic}}$(V) is the ohmic overpotential and $\eta_{\text{mass transport}}$(V) is the mass transport overpotential. An empirical equation developed by Leroy et al. is adopted to determine the reversible potential:

$E_{\text{rev}}=1.5184-1.5421\times10^{-3} T+9.523\times10^{-5} T lnT+ 9.84\times10^{-8} T^{2}\text{ }$ (2)

Where *T*(K) is the temperature. The ohmic overpotential can be calculated by Ohm's law:

$\eta_{\text{ohmic}}=i R_{\text{ohmic}}\text{ }$ (3)

where $R_{\text{ohmic}}$ is obtained from the HFR, and $i$(A cm^-2^) is the current density. The activation overpotential is approximated by the following equation:

$\eta_{\text{activation}}=b log_{10}\left(\frac{i}{i_{0}}\right)$ (4)

where $b$(mV dec^-1^) and $i_{0}$(A cm^-2^) represent the Tafel slopes and apparent exchange current density obtained through Tafel analysis. According to Eq. (1), the mass transport overpotential $\eta_{\text{mass transport}}$ can be derived by subtracting other variables from the cell voltage$E_{\text{cell}}$.

***High-Speed visualization system***

The Revealer M230M high-speed camera coupled with microscale lens were used to visualize millimeter-scale two-phase flow in the flow field ^[13]^. In this study, a recording frame rate of 1000 fps and exposure of 500 were chosen to record the two-phase flow in flow field. Six videos about two-phase flow in the flow field pre- and post-durability were captured at the current densities of 0.5 A cm^-2^, 1.0 A cm^-2^ and 3.0 A cm^-2^, As for the micrometer-scale bubble behaviors at the electrode surfaces, the Revealer M230M high-speed camera coupled with microscope were used to realize very clear observations, as shown Figure S1. A recording frame rate of 1000 fps and exposure of 5000 was chosen to record the bubble behaviors. Two videos about bubble behaviors at the electrode surfaces pre- and post-durability were captured at the current densities of 500 mA cm^-2^. It was a three-electrode electrochemical measurement performed in a square H-cell.

***Three electrode measurements***

Three electrode testing was conducted using 1.5 cm × 1.5 cm stainless steel felts pre- and post-electrodes as the working electrode, an Hg/HgO electrode as the reference, and a Pt sheet (1.5 cm × 1.5 cm) as the counter electrode in 1.0 M KOH at room temperature. The two electrodes were each operated at a constant current of 500 mA cm^-2^.

**Figures**


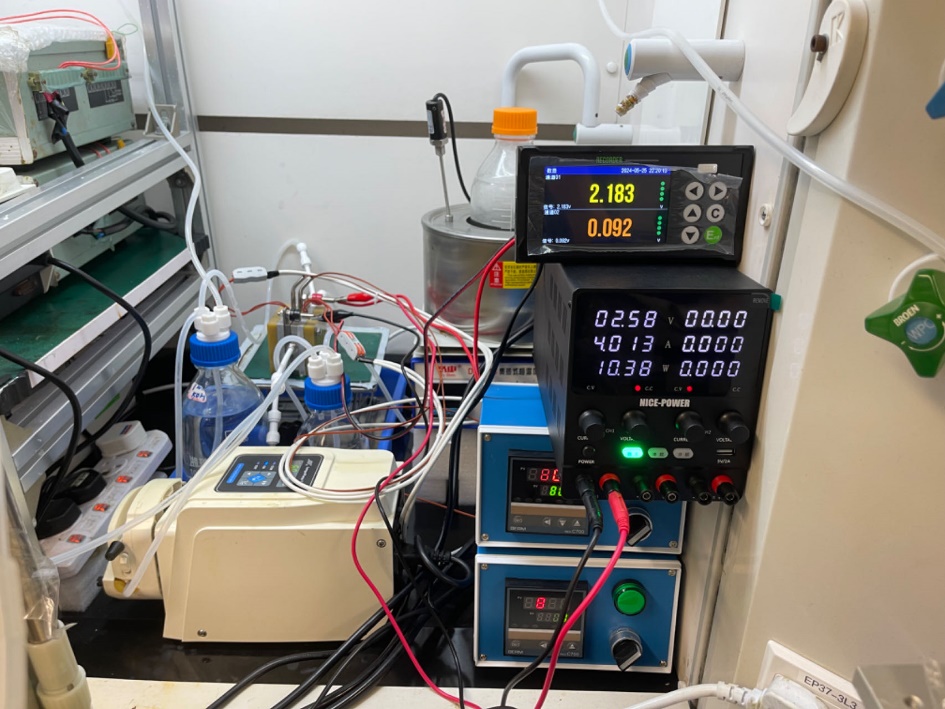


Figure S1. Setup for durability test.


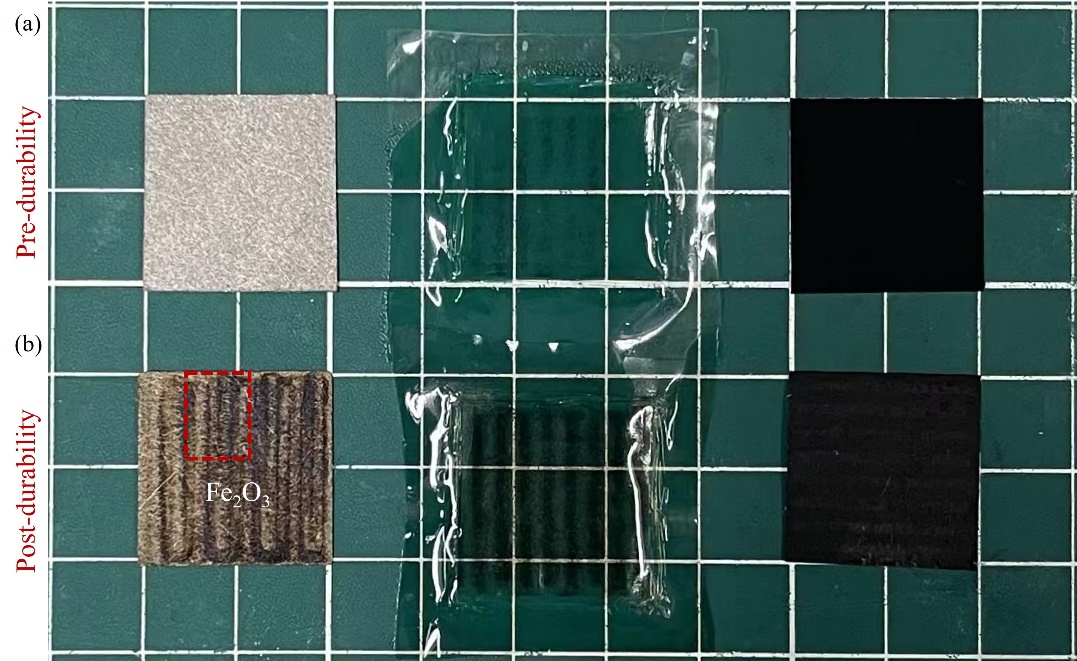


Figure S2. MEA pre-and post-durability.


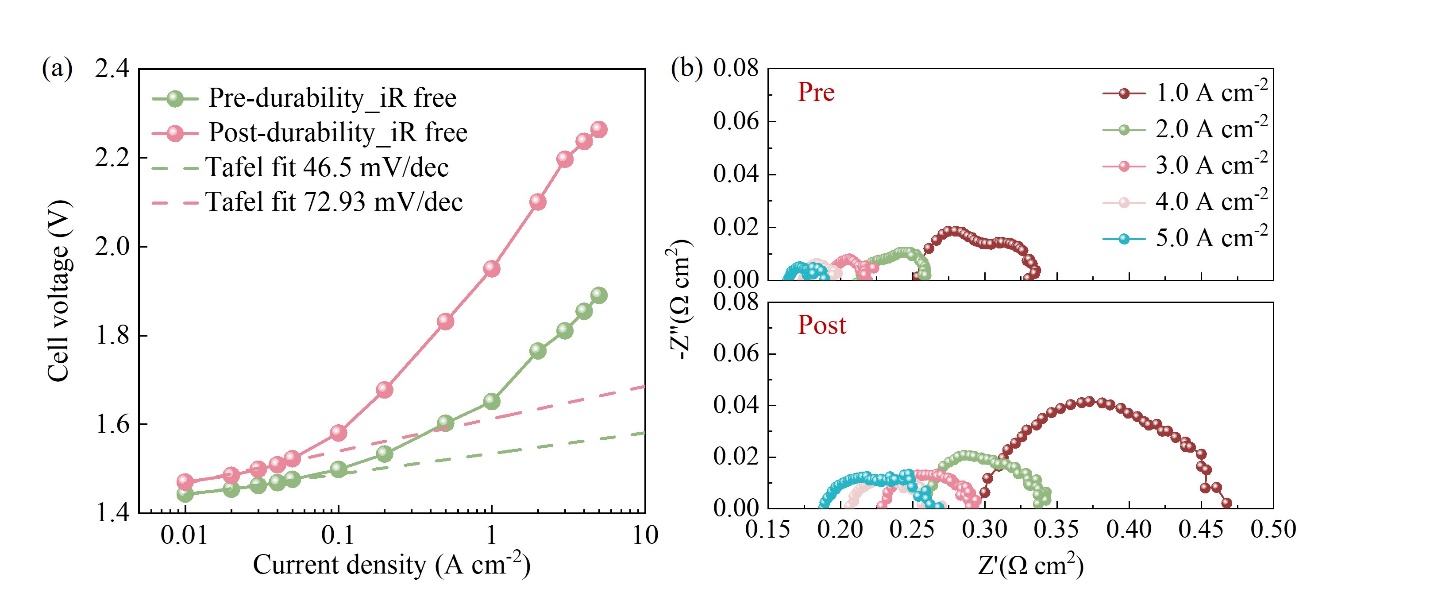


Figure S3. Electrochemical performance of the AEMWE pre-and post-durability: (a) Tafel slope and iR-free cell voltage, (b) high-frequency resistance at different current densities.


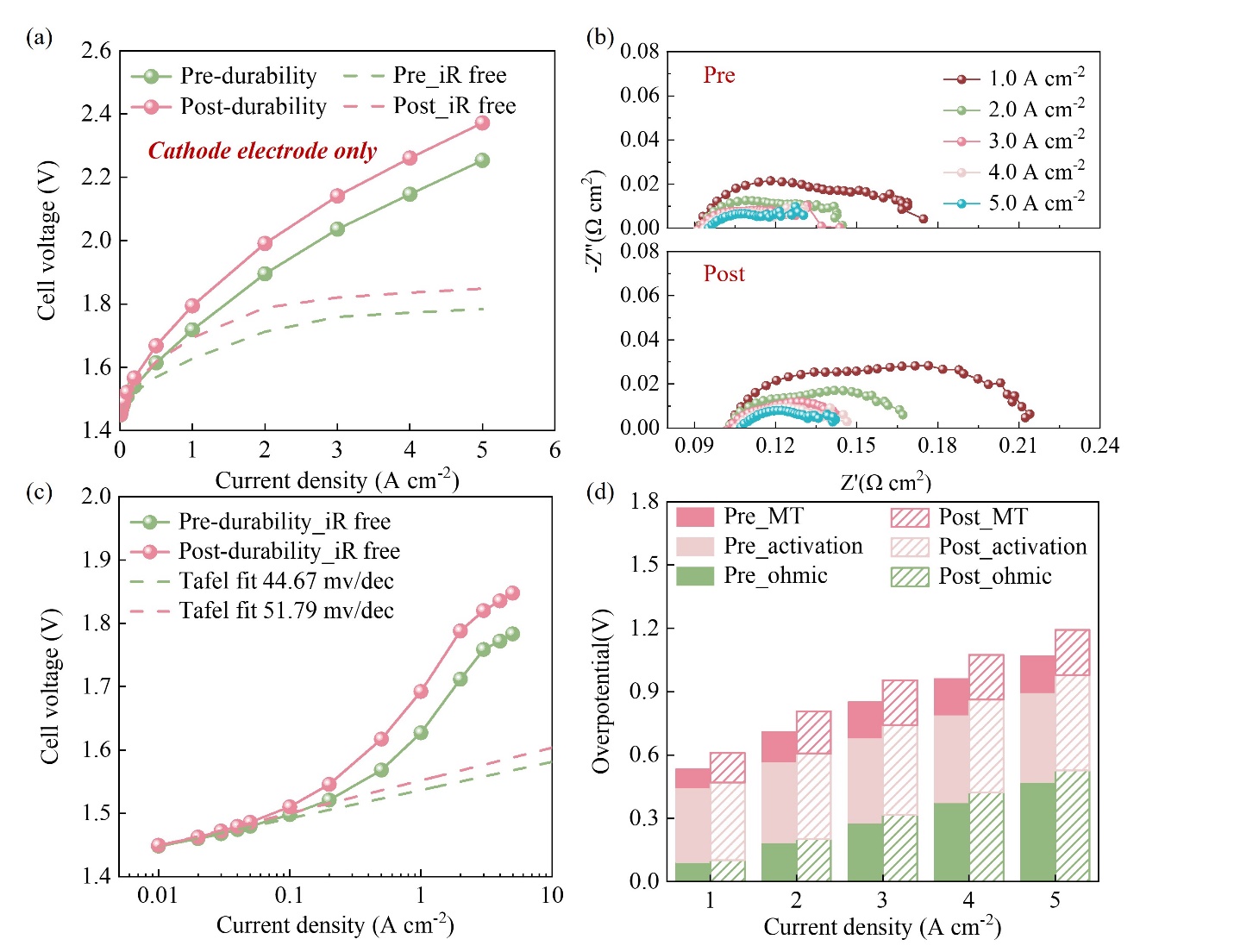


Figure S4. Electrochemical performance of the AEMWE with new membrane, new anode electrode, new cathode electrode and new membrane, new anode electrode, post-durability cathode electrode: (a) polarization curves, (b) high-frequency resistance at different current densities, (c) Tafel slope and iR-free cell voltage, and (d) breakdown of overpotential at different current densities.

*
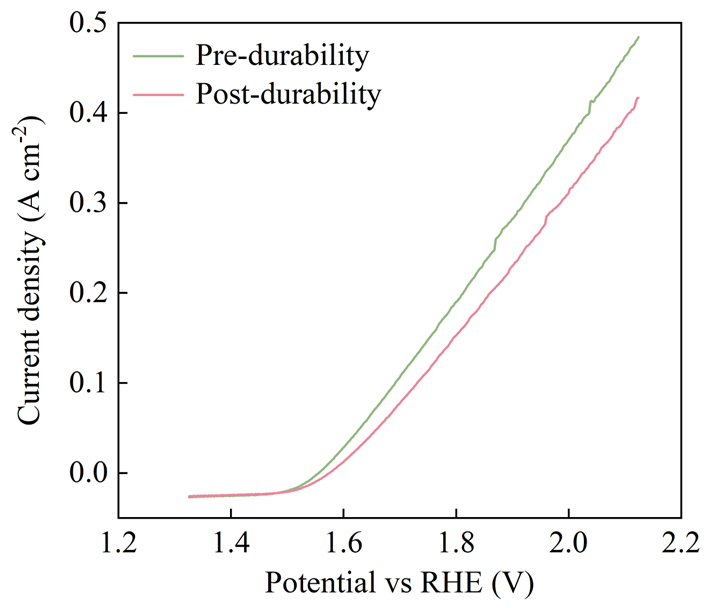
*

Figure S5. Linear sweep voltammetry measurements of the pre- and post-durability electrode*.*


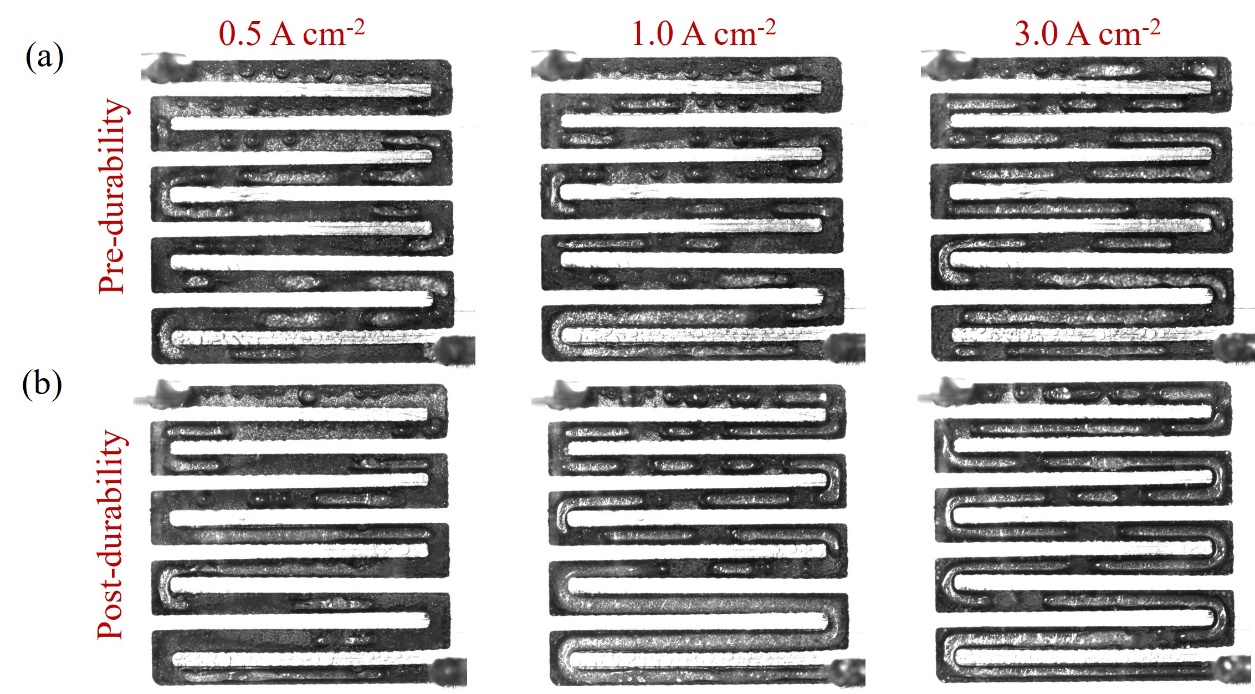


Figure S6. Two-phase flow visualization in flow field at current densities of 0.5 A cm^-2^, 1.0 A cm^-2^ and 3.0 A cm^-2^.


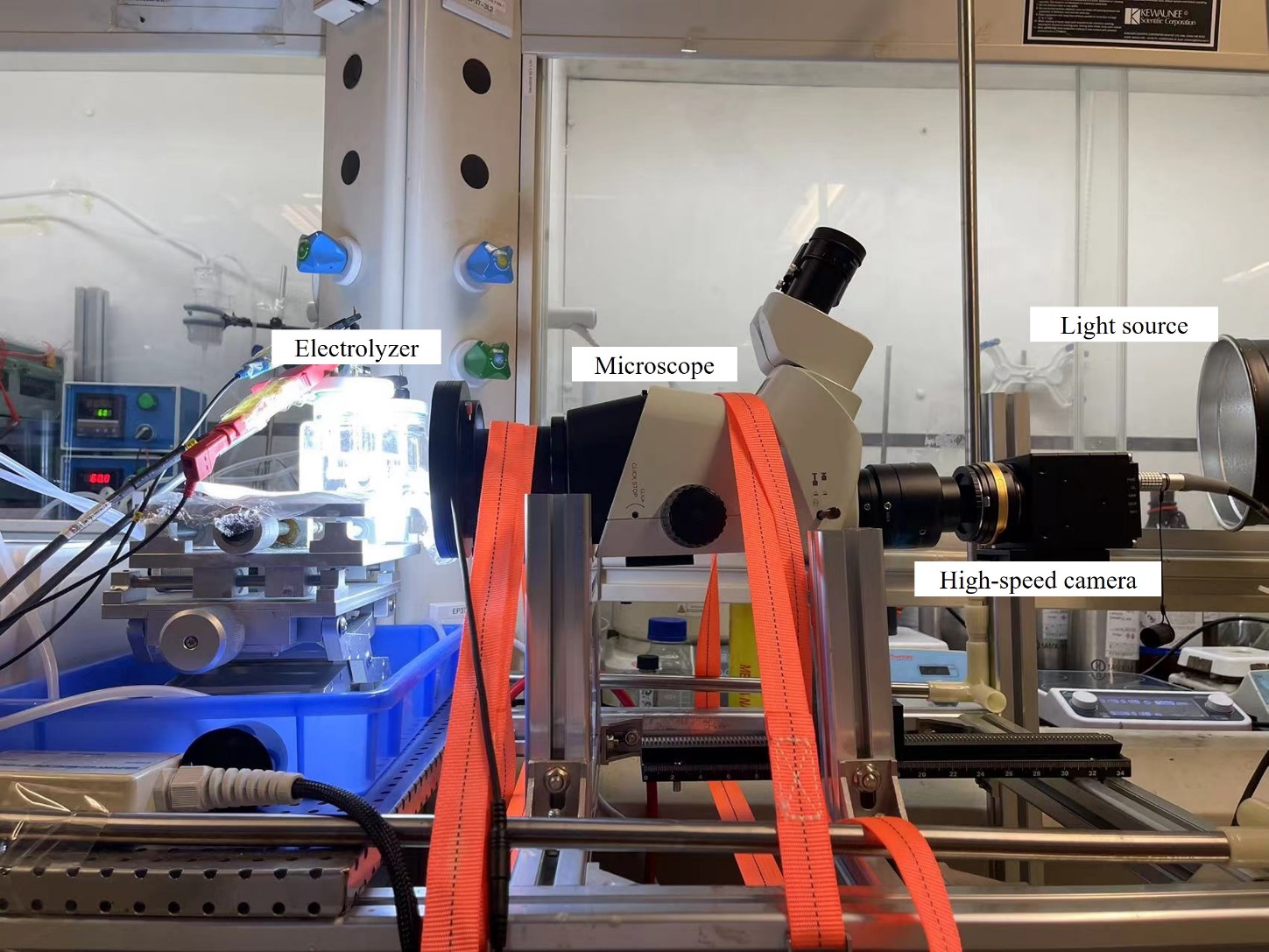


Figure S7. High-speed visualization system for micrometer-scale bubbles.

.


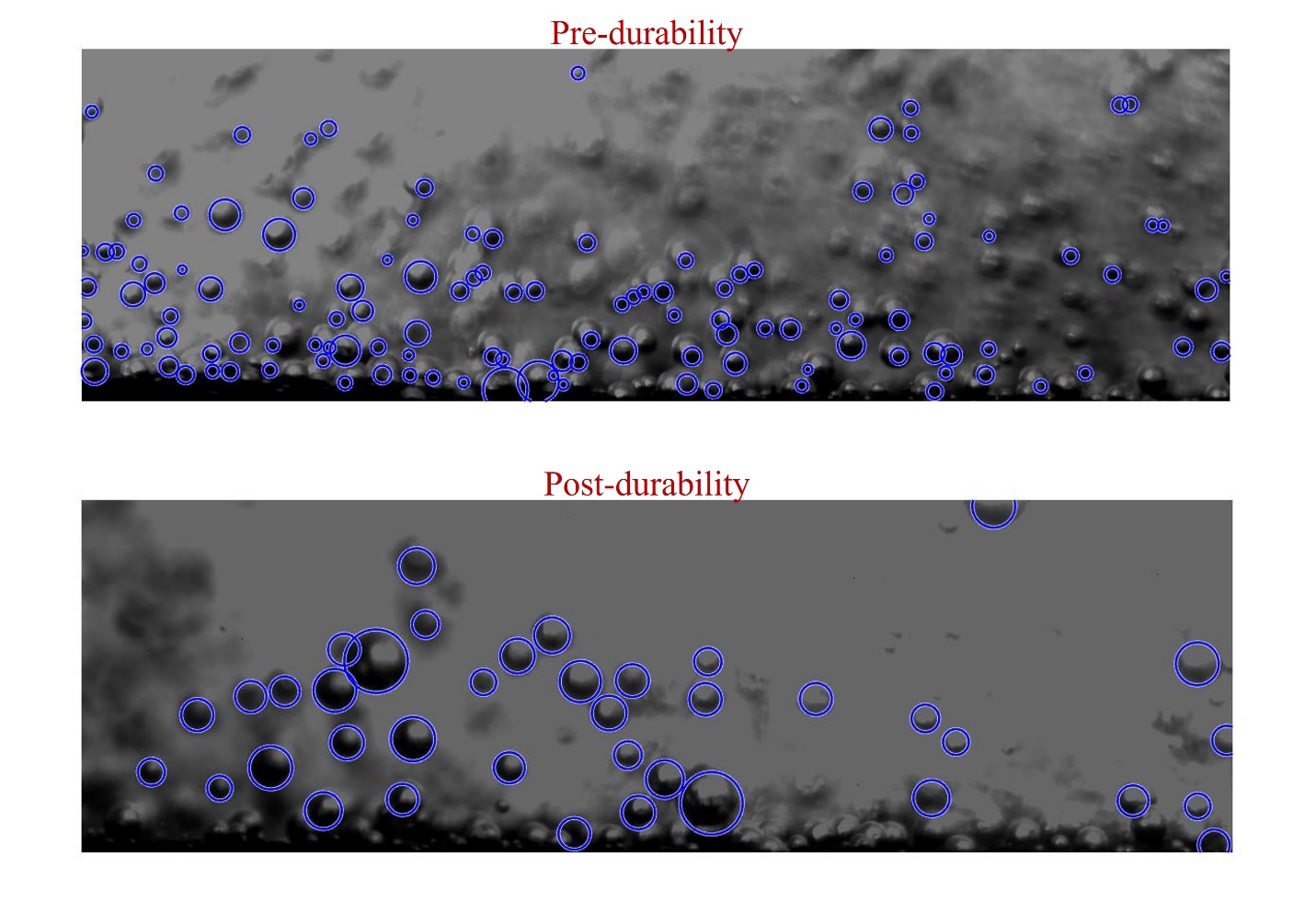


Figure S8. Bubble recognition process.

**References**

(1) Li, W.; Liu, Y.; Zhang, Z.; Pan, Z.; Chen, R.; An, L. Performance of a Hybrid Direct Ammonia Fuel Cell with Hydrogen Peroxide Reduction. *J. Power Sources* **2024**, *593*, 233985.

[2] Z. Pan, F. Xie, Z. Zhang, Z. Zhao, L. Wu, W. Li, Y. Zhu, X. Huo, Y. Liu, X. Zhang, R. Chen, L. An, *Adv. Funct. Mater.* **2024**, 2404710.

[3] Z. Pan, L. Wu, F. Xie, Z. Zhang, Z. Zhao, O. C. Esan, X. Zhang, R. Chen, L. An, *Energy AI* **2024**, 100400.

[4] Z. Pan, Z. Zhang, W. Li, X. Huo, Y. Liu, O. C. Esan, Q. Wu, L. An, *ACS Energy Lett.* **2023**, *8*, 3742.

(5) Zuo, Y.; Mastronardi, V.; Gamberini, A.; Zappia, M. I.; Le, T.-H.-H.; Prato, M.; Dante, S.; Bellani, S.; Manna, L. Stainless Steel Activation for Efficient Alkaline Oxygen Evolution in Advanced Electrolyzers. *Adv. Mater.* **2024**, 2312071.

(6) Tricker, A. W.; Ertugrul, T. Y.; Lee, J. K.; Shin, J. R.; Choi, W.; Kushner, D. I.; Wang, G.; Lang, J.; Zenyuk, I. V.; Weber, A. Z.; Peng, X. Pathways Toward Efficient and Durable Anion Exchange Membrane Water Electrolyzers Enabled By Electro-Active Porous Transport Layers. *Adv. Energy Mater.* **2024**, *14* (9), 2303629.

[7] B. Chen, A. L. G. Biancolli, C. L. Radford, S. Holdcroft, *ACS Energy Lett.* **2023**, 2661.

[8] A. W. Tricker, J. K. Lee, J. R. Shin, N. Danilovic, A. Z. Weber, X. Peng, *J. Power Sources* **2023**, *567*, 232967.

[9] W. Wang, K. Li, L. Ding, S. Yu, Z. Xie, D. A. Cullen, H. Yu, G. Bender, Z. Kang, J. A. Wrubel, Z. Ma, C. B. Capuano, A. Keane, K. Ayers, F.-Y. Zhang, *ACS Appl. Mater. Interfaces* **2022**, *14*, 9002.

[10] B. Zhao, C. Lee, J. K. Lee, K. F. Fahy, J. M. LaManna, E. Baltic, D. L. Jacobson, D. S. Hussey, A. Bazylak, *Cell Rep. Phys. Sci.* **2021**, *2*, 100580.

(11) Kim, P. J.; Lee, J. K.; Lee, CH.; Fahy, K. F.; Shrestha, P.; Krause, K.; Shafaque, H. W.; Bazylak, A. Tailoring catalyst layer interface with titanium mesh porous transport layers. *Electrochim. Acta* **2021**, *373*, 137879.

[12] S. Yuan, C. Zhao, X. Mei, S. Shen, Q. Wang, X. Yan, J. Zhang, *Int. J. Heat Mass Transf.* **2023**, *212*, 124249.

[13] L. Wu, Z. Pan, S. Yuan, X. Shi, Y. Liu, F. Liu, X. Yan, L. An, *Chem. Eng. J.* **2024**, *488*, 151000.
